# A SYSTEMATIC REVIEW OF FUNCTIONAL TRAINING EXERCISE IN COMMUNITY-DWELLING OLDER ADULTS WITH COGNITIVE IMPAIRMENT

**DOI:** 10.1093/geroni/igad104.2303

**Published:** 2023-12-21

**Authors:** Yun Chan Shin, Chiung-ju Liu, Kellen Breton, Justin Mason, Yi-Ling Hu, Jane Morgan-Daniel

**Affiliations:** University of Florida, Gainesville, Florida, United States; University of Florida, Gainesville, Florida, United States; University of Florida, Gainesville, Florida, United States; University of Iowa, Coralville, Iowa, United States; Chang Gung University, Kwei-Shan, Taoyuan, Taiwan (Republic of China); University of Florida, Gainesville, Florida, United States

## Abstract

Older adults with cognitive impairment are at risk of becoming care dependent. Functional training involves movements necessary for daily tasks, which may help maintain or improve independence. As part of a larger systematic review examining the effects of a functional training exercise in community-dwelling older adults, this study aimed to synthesize the functional and cognitive outcomes in those with cognitive impairment. A systematic search was conducted in 10 electronic databases to identify relevant studies published between January 2010 and September 2021. Studies were selected if recruited adults aged > 60 years, participants had a cognitive impairment that was not due to acquired brain injury, and activities of daily living and/or cognitive function were measured. Nine studies (847 participants) were identified. Five studies were conducted on older adults with mild cognitive impairment, all showed an improvement in activities of daily living, and 4 showed an improvement in global cognition. Three were conducted on older adults with dementia, and one showed an improvement in activities of daily living but not global cognition. One was conducted on older adults with mild cognitive impairment or dementia and reported an improvement in both outcomes. Functional training exercise seems effective in improving cognitive function and activities of daily living in older adults with mild cognitive impairment. For those with dementia, although research on functional training is limited, some preliminary evidence suggests that incorporating functional training with other components may be beneficial. Despite inconclusive findings, functional training exercises may hold potential for older adults with cognitive impairment.

